# Diagnostic Performance of F-18 FDG PET/CT in the Detection of Recurrent Colorectal Cancer: Correlation with Biochemical Markers and Conventional Imaging Modalities

**DOI:** 10.3390/jcm13123602

**Published:** 2024-06-20

**Authors:** Jasna Mihailović, Jelena Roganović, Ivana Starčević, Ivan Nikolić, Nataša Prvulović Bunović, Zoran Nikin

**Affiliations:** 1 Department of Nuclear Medicine, Faculty of Medicine, University of Novi Sad, Hajduk Veljkova 3, 21000 Novi Sad, Serbia; prvulovic.natasa@onk.ns.ac.rs; 2 Division of Nuclear Medicine, Oncology Institute of Vojvodina, Put dr Goldmana 4, 21204 Sremska Kamenica, Serbia; roganovic.jelena@onk.ns.ac.rs (J.R.); istarcevic97@gmail.com (I.S.); 3 Department of Internal Medicine, Faculty of Medicine, University of Novi Sad, Hajduk Veljkova 3, 21000 Novi Sad, Serbia; nikolic.ivan@onk.ns.ac.rs; 4 Clinic for Medical Oncology, Oncology Institute of Vojvodina, Put dr Goldmana 4, 21204 Sremska Kamenica, Serbia; 5 Centre for Diagnostic Imaging, Oncology Institute of Vojvodina, Put dr Goldmana 4, 21204 Sremska Kamenica, Serbia; 6 Department for Pathoanatomical Diagnostics, Oncology Institute of Vojvodina, Put dr Goldmana 4, 21204 Sremska Kamenica, Serbia; nikin.zoran@onk.ns.ac.rs

**Keywords:** colorectal cancer, recurrence, detection, F-18 FDG PET/CT, CEA, CA 19-9, conventional imaging

## Abstract

**Background/Objectives**: Although the role of PET/CT imaging is well established in oncology, its diagnostic value in routine monitoring for recurrent colorectal cancer (CRC) is still controversial. The aim was to evaluate the diagnostic value of F-18 FDG PET/CT in detecting recurrent CRC in correlation with CEA, CA 19-9 levels, and conventional imaging modalities (CIM). **Methods:** Between 2009 and 2023, a retrospective study was performed including 134 CRC patients referred for PET/CT imaging on the suspicion of recurrence, based on elevated CEA and/or CA 19-9 and/or equivocal CIM findings. According to our institution’s Tumor Board CRC protocol, after the initial treatment, which was dependent on the TNM stage (neoadjuvant therapy, primary resection, or adjuvant treatment), patients underwent a standard 5-year surveillance including CEA and CA 19-9 measurements, CIM, and colonoscopy, every six months. The statistics, including univariate and multivariate analyses were conducted using the IBM SPSS 20.0 statistical software. *p*-values < 0.05 were considered statistically significant. **Results:** Recurrent CRC was confirmed in 54/134 (40.3%) patients with elevated tumor markers. PET/CT showed high diagnostic performance in detecting recurrent CRC with sensitivity, specificity, PPV, NPV, and accuracy of 94.4%, 82.5%, 78.5%, 95.7%, and 87.3%, respectively. The CEA showed a high sensitivity of 98.1% but both low specificity and accuracy of 15% and 48.5%, respectively. The sensitivity, specificity, and accuracy for CA 19-9 and CIM for diagnosis of CRC recurrence were 44.4%, 67.5%, 58.2%, and 51.9%, 98.8%, 79.9%, respectively. The AUC for PET/CT, elevated CEA levels, CIM, and elevated CA 19-9 levels was 0.885 (95% CI: 0.824–0.946; *p* < 0.001), 0.844 (95% CI: 0.772–0.916; *p* < 0.001), 0.753 (95% CI: 0.612–0.844; *p* < 0.001), and 0.547 (95% CI: 0.442–0.652; *p* = 0.358), respectively. Univariate analysis showed that both PET/CT and CIM positive results were highly associated with CRC recurrence (*p* < 0.001 and *p* < 0.001, respectively). At the same time, gender, mucinous tumor type, presence of initial lymph node metastasis (N+), and presence of initial distant metastasis (M+) had no significance (*p* = 0.211, *p* = 0.158, *p* = 0.583, and *p* = 0.201, respectively). Our multivariate analysis showed that independent predictors for CRC recurrence are positive PET/CT scans (*p* < 0.001), positive CIM results (*p* = 0.001), and elevated CA 19-9 levels (*p* = 0.023). Although CA 19-9 was not detected as a statistically significant predictor in the univariate analysis (*p* = 0.358), in a multivariate analysis it was recognized as a significant predicting factor in detecting the CRC recurrence (*p* = 0.023). **Conclusions:** F-18 FDG PET/CT showed high diagnostic efficacy in CRC recurrence detection, in correlation with CEA levels, CA 19-9 levels, and CIM. This imaging modality should be routinely integrated into the post-operative follow-op in patients with elevated tumor markers.

## 1. Introduction

Colorectal cancer (CRC) is one of the most frequent malignancies in the human population. In the current year, the latest United States cancer statistics data estimate new CRC cases accounting for 8% of male population and 7% of female population. In addition, CRC has a high mortality rate, with 9% of estimated deaths in men and 8% in women for the same year [1]. Radical tumor resection is the initial treatment for the majority of non-metastatic CRC patients, which improves long-term survival. Additional adjuvant or neoadjuvant chemoradiotherapy, and combination treatments, depend on the patient’s staging and the aim to provide better outcomes [2,3]. Recurrence rates have been reported in 30–50% of patients during the post-operative monitoring, depending on the stage [4,5,6,7], with the highest recurrence rate within the first two years [8]. However, recent reports show a declining incidence of CRC recurrence of 15–16% [9,10] and 21–23% [6,7], mostly due to better screening programs and improved treatment options.

Since relapsed CRC has a poor prognosis, early detection of recurrence is essential. The European Society for Medical Oncology suggests non-metastatic CRC patient monitoring five years post-initial surgery, including carcinoembryonic antigen (CEA) level determination, computed tomography (CT), and a colonoscopy [11,12]. Current guidelines for surveillance of CRC patients within 3 or 5 years post-initial treatment recommend regular and periodical CT and CEA testing, while frequency depends on the stage and risk for recurrence [13,14,15].

Besides the CEA, there are some other tumor markers which may also be performed in clinical practice such as carbohydrate antigen (CA 19-9), tissue polypeptide specific antigen (TPS), cancer antigen 125 (CA 125), serum ferritin (SF), tumor-associated glycoprotein-72 (TAG-72), and hematopoietic growth factors (HGF-s) [16,17]. Hence, the CEA is considered the most effective test in detecting recurrent CRC. According to the ASCO and ESMO associations, it is a golden standard for monitoring CRC with regular evaluations every three months during the first three years, and every six months for the subsequent 2–3 years [18,19].

Standard imaging modalities performed for the early detection of recurrences include CT and magnetic resonance imaging (MRI). Although the PET/CT has been shown as a valuable technique in detecting CRC recurrence, its role is still controversial. This hybrid imaging, i.e., molecular imaging, is a non-invasive diagnostic modality that can distinguish between malignant and benign lesions based on the differences in their metabolic activities [20]. Despite its ability to provide data on tumor metabolism and whole body image in one exam, the role of metabolic imaging in detecting CRC recurrence is still a matter of debate. Currently, the PET/CT is not integrated into the NCCN guidelines as a routine monitoring method for the recurrent CRC. However, in patients with elevated CEA, PET/CT is an efficient method for post-operative detection of recurrent and metastatic CRC [21,22].

This study aimed to evaluate the diagnostic accuracy of F-18 FDG PET/CT in detecting recurrent colorectal cancer and to compare it with conventional imaging and tumor markers, CEA and CA 19-9.

## 2. Materials and Methods

### 2.1. Patients

In this retrospective study of observational nature, 134 CRC patients with increased tumor markers (either only CEA, CA 19-9, or both) underwent PET/CT imaging in our institution on suspicion of recurrent CRC. Our study was performed between 2009 and 2023 during the patient’s follow-up.

The inclusion criteria were as follows: (a) histopathological diagnosis of CRC, (b) all patients achieved remission of disease after the initial treatment, (c) recurrence suspicion due to at least two or several elevated levels of CEA and/or CA 19-9 above the upper normal threshold value, and (d) results obtained by conventional imaging modalities (CIM) and/or colonoscopy. Patients who had another type of malignancy were not included in the study.

The management of CRC in our institution follows the institution’s Tumor Board protocol. The treatment algorithm is specifically designed for each patient (personalized treatment approach). Patients with a tumor localized in the colon underwent primary tumor resection with or without adjuvant chemotherapy, depending on the TNM stage. Patients with rectal cancer underwent a tumor resection only, neoadjuvant treatment (chemoradiation) before surgery, or chemotherapy following initial treatment, depending on the histological report. After the initial treatment was completed, patients were regularly monitored. Our 5-year follow-up protocol for CRC includes measurements of CEA and CA 19-9, conventional imaging modalities (CT and MRI), and colonoscopy periodically at six months. Patients were referred to PET/CT imaging in case of any sign of recurrence, including elevated levels of CEA and/or CA 19-9 and/or inconclusive CT and/or MRI results or inconclusive results of colonoscopy.

### 2.2. Protocol

The final diagnosis of CRC recurrence was confirmed using the reference (gold) standard, a histological report after surgical resection/biopsy or clinical follow-up, including tumor marker measurements, CIM, and colonoscopy for at least six months after the PET/CT scan. CIM was performed including CECT and/or MRI depending on indications and availability.

Imaging results, as well as clinical, histopathological, and laboratory data were collected from patients’ medical files. The Oncology Institute of Vojvodina Ethics Committee from Sremska Kamenica approved this study.

### 2.3. Biochemical Markers

The institutional Tumor Board’s protocol recommends a routine 5-year surveillance of CRC patients with measurements of both CEA and CA 19-9. Serum CEA and CA 19-9 measurements were carried out by an automatic analyzer that uses immune-enzymatic assays (ECLIA, Roshe, Cobas e 411). Normal serum values of CEA are <4.70 ng/mL, while normal values for CA 19-9 are <39.0 U/mL. Two consecutive increased values of either CEA or CA 19-9, or both, were considered suspicious of having recurrent CRC.

### 2.4. Data Acquisition, Reconstruction, and Image Interpretation

In our institution, PET/CT imaging was performed on a 64-slice hybrid PET/CT scanner (Biograph, TruePoint 64, Siemens Medical Solutions, Knoxville, TN, USA) from 2007 until the end of June 2022, when this machine was replaced by a new PET/CT scanner (Discovery MI, DR, GE Medical Systems LLC, Milwaukee, WI, USA) in July of the same year.

Patients fasted for at least 6 h before the PET/CT exam. The automatic injector injected an F-18 FDG of 3.7 MBq/Kg intravenously. Acceptable glucose levels for PET/CT scan include values of less than 11 mmol/L in all patients. After the radiotracer administration, patients rested in basal conditions for 60–90 min. During this uptake phase, patients had to drink one liter of oral contrast dispersion which helped to better delineate the lymph nodes and a normal bowel. After resting, patients underwent a PET/CT exam.

PET/CT imaging at Biograph included non-enhanced CT scans that were acquired with 120 kV, 40 mA, slice thickness 5 mm, pitch 1.5, and rotation time 0.5 s for topographic localization and attenuation correction. Subsequent PET acquisition was carried out using 3 min per bed and 6–7 beds per patient in three-dimensional mode. Non-corrected and attenuation-corrected CT, PET, fused PET/CT images, and MIP were analyzed on the Syngo Multimodality Workstation, Siemens AG, Munich, Germany.

PET/CT imaging at Discovery includes a 128 slice-CT (Revolution EVO) performed in a craniocaudal direction. The first scout (i.e., topogram) was acquired with 120 kV, 10 mA, and a scout plane 180. Low-dose CT for attenuation was acquired with 64 × 0.625 mm detectors and 40 mm beam collimation [120 kV, 250 mA, in 0.5 s with 512 matrices]. The Display Field Of View (DFOV) was 70 cm. Data were reconstructed using a 2.5 mm standard recon type with ASIR 60%. Soon after the CT scanning ended, the PET scan was immediately acquired in a three-dimensional mode, from the base of the skull to mid-thigh, at 2.2 min per bed position (total of beds depending on patient’s height), 256 matrices, and FOV of 150 mm. The CT data were matched and fused with the PET data. Reconstruction was performed using the ordered subsets’ expectation maximization 3D reconstruction method, with VPFX cut-off filter 5 mm, 24 subsets, and 2 iterations. Data from both CT and PET were sent to the AW server workstation, with installed PET options (GE Healthcare) for clinical evaluation.

A positive PET/CT scan was considered when a pathological uptake (focal FDG uptake area more significant than the background) was detected, after the physiologically increased uptake was excluded, with or without corresponding CT lesion. Lesions were analyzed qualitatively and semi-quantitatively. Quantitative measurements of FDG uptake in the lesions were based on the maximum standardized uptake value per focus (SUVmax). This value was calculated at tissue activity (expressed as counts/pixel/s) multiplied by the calibration factor divided by injected 18F-FDG dose (MBq/kilogram of body weight). No absolute cut-off value was used for the diagnosis. Tumor lesions were defined by the volume of interest (VOI) at lesions with a high FDG uptake, with a 50% threshold.

Three experienced physicians (two nuclear medicine physicians and one radiologist) blindly reviewed all PET/CT images and provided diagnoses separately. The patients were thereby classified as positive or negative for CRC recurrence. In cases of discrepancy, the PET/CT images were re-examined and a consensus reached a definitive diagnosis. Using the nuclear medicine workstation, the maximum ROIs were drawn over the lesions detected as having the most intense uptake. The highest SUVmax values identified for each patient were selected.

True-positive lesions were presented with positive tumor markers, CIM, and PET/CT, confirmed for malignancy by the reference standard (histology or follow-up). Lesions were true negative if negative tumor markers, CIM, and PET/CT results were confirmed by histology or by decreasing CEA levels to normal values without imaging evidence of recurrence during the subsequent follow-up. False-positive cases include suspicious lesions with confirmed negative histology for malignancy or lesions that had resolved by the time of follow-up imaging. Lesions were false negative if tumor markers, CIM, or PET/CT results were negative, but the reference standard confirmed malignancy.

### 2.5. Statistical Analysis

Numbers and percentages were used to describe categorical data; mean, median, standard deviation, IQR, minimum, and maximum values were used for continuous data. The association between clinical and pathological variables and CRC recurrence was performed with the Mann–Whitney U test (CEA and Ca 19-9 levels), while Fisher’s exact test was used for categorical data.

Receiver operating characteristic (ROC) analysis was used to assess the accuracy of model predictions by plotting sensitivity versus 1-specificity. The area under the ROC curve (AUC) was used to evaluate the accuracy of different methods (PET/CT, CIM, CEA, CA 19-9) in detecting CRC recurrence. ROC curve analysis was applied to identify the best-discriminating cut-off values of CEA and CA 19-9 between patients with recurrence and patients free of disease. The following diagnostic quality parameters were calculated: sensitivity (SN), specificity (SP), positive predictive value (PPV), negative predictive value (NPV), false-positive value (FPV), false-negative value (FNV), and overall accuracy (ACC) for PET/CT, CIM, and tumor markers including CEA and CA 19-9 for the detection of recurrent CRC.

To find independent predictors for CRC recurrence, multivariate analysis (binary logistic regression model—forward stepwise conditional) was used. The statistical analysis was conducted using IBM SPSS 20.0 statistical software. *p*-values below 0.05 were considered statistically significant.

## 3. Results

### 3.1. Patients’ Demographic, Clinical and Histopathological Data

This retrospective study was conducted on 134 patients (mean age 69.6 ± 11.0 years; range 39–89 years). The study included 79 (59%) males and 55 (41%) females, with a mean age of 70.6 ± 11.3 years and 68.2 ± 10.5 years, respectively. All 134 patients had histologically proven CRC, including colon cancer in 99 patients and rectal cancer in 35 patients. Colon cancer was detected at different localizations, including ascending colon (17 patients), caecum (11 patients), transversal colon (12 patients), descending colon (10 patients), sigmoid colon (25 patients), rectosigmoid colon (13 patients), splenic flexure in 6 patients, and liver flexure in 3 patients. CRC presented as a single tumor in 132 patients or as a duplex cancer with combined sites in two patients. Out of 134 patients with CRC adenocarcinoma, 22 patients had the mucinous tumor type. The remaining 112 patients with CRC adenocarcinoma were divided into several tumor differentiation grades. The classification of tumor types, differentiation (i.e., tumor grade), and initial TNM staging were determined according to the current classifications [23,24]. The patients’ demographic, clinical, and histopathological characteristics have been summarized in Table 1.

### 3.2. Diagnostic Methods in Detecting CRC Recurrence

Recurrent CRC was diagnosed in 54 patients as a positive result by all four performed methods (PET/CT, CIM, elevated CEA, and elevated CA 19-9) in 9 patients (16.7%). Imaging methods, including both PET/CT and CIM, were positive in 24 (44.4%) of patients; positive PET/CT scans but negative CIM results were obtained in 27 (50.04%) patients, while positive CIM but negative PET/CT scans were detected in only 3 (5.8%) of patients. Among 54 CRC recurrences, elevated levels of both CEA and CA 19-9 were detected in 22 (40.7%) patients; elevated CEA levels but normal CA 19-9 levels were in 31 patients (57.4%); and elevated CA 19-9 levels but normal CEA were only in 1 patient (1.9%).

#### 3.2.1. Imaging Modalities

The recurrent CRC was confirmed in 54 (40.3%) patients, histolopathologically in 15/54 (27.8%) patients, after surgery in 13 patients, and after a biopsy of the suspicious lesion in 2 patients. In the remaining 39/54 (72.2%) patients, a definitive diagnosis was based on subsequent follow-up (including CIM, colonoscopy, and clinical/laboratory results). Localization of the recurrent CRC and methods for assessing the final diagnosis are shown in Table 2. Nodal involvement was detected in 11/54 patients, while local, peritoneal, and locoregional recurrence was found in 7/54 patients, 5/54 patients, and 4/54 patients, respectively. Distant metastases occurred in the liver and lungs in nine and six patients, respectively. CRC recurrence was detected both in the liver and lungs in 6/54 patients.

CIM (CECT, MRI, or both) provided correct diagnosis in 107 patients, with true-positive results in 28 patients and true-negative results in 79 patients. Twenty-seven patients were misdiagnosed by CIM, including false-negative results in twenty-six patients and false-positive results in one patient.

PET/CT provided correct diagnosis in 117 out of 134 patients, with true-positive results in 51 patients and true-negative results in 66 patients. Based on both PET/CT and CIM, the results were positive in 25/51 cases, 16/51 patients received subsequent chemotherapy, 6/51 patients underwent surgery, while the remaining three patients received surgery followed by chemotherapy. In addition, PET/CT positive results altered subsequent patients’ management in 26 CIM false-negative results—22/26 patients subsequently received chemotherapy (one of those previously underwent excisional biopsy of local recurrence) while the remaining 4/26 patients underwent surgical resection of distant metastases [liver resection in three patients (two out of three patients underwent surgery, while the remaining patient with combined liver + peritoneal recurrence received liver resection followed by chemotherapy), with one patient undergoing lung surgery].

Seventeen patients were misdiagnosed by PET/CT results, including three patients with false-negative PET/CT results and fourteen patients with false-positive PET/CT scans. CRC recurrence was confirmed in three patients with false-negative PET/CT results by a subsequent imaging follow-up: one patient with a mucinous adenocarcinoma underwent MR imaging, which detected a bone metastasis in a right pelvic bone, while a CECT scan confirmed peritoneal lesion in one and a local recurrence in another patient. Subsequent treatment, including chemotherapy, was performed in all of these three patients after the confirmation of a CRC recurrence. PET/CT scans show false-positive results in 14/134 patients with CRC, according to the hypermetabolic lesions detected at different sites: lymph nodes (8/14 patients), locoregional (2/14 patients), lungs (2/14 patients), and liver (2/14 patients). In 2/14 patients, suspected nodal involvement was histologically confirmed as negative after a biopsy. In comparison, the absence of recurrent CRC was confirmed in the remaining 12/14 patients based on the follow-up algorithm including decreasing tumor markers and negative colonoscopy and/or CIM results.

PET/CT was able to detect recurrent CRC locally (6/54 patients) in regional lymph nodes (11/54 patients), locoregionally (4/54 patients), peritoneum (4/54 patients), liver (9/54 patients), lungs (6/54 patients), and in combined sites: liver and lungs (6/54 patients), liver and peritoneum (4/54 patients), and liver, lungs, and bone (1/54 patient). PET/CT detected CRC recurrence in the liver in nine patients, with elevated CEA in eight out of nine patients; elevated CEA and CA 19-9 in three out of nine patients, while false-negative results of CEA and CA 19-9 were detected in one out of nine and five out of nine patients, respectively. In seven out of nine patients, PET/CT scan agreed with MRI, while the remaining two out of nine patients had false-negative CT scans. PET/CT was able to detect distant metastases in 26 out of 54 CRC patients with recurrence, which were located in lungs, liver and combined sites. Among the patients, liver metastases were identified in 9 out of 54 cases. Subsequent treatment included liver resection in six patients and chemotherapy in three patients. PET/CT detected lung metastases in 6/54 patients who underwent the following treatment—chemotherapy in three patients, surgery in two patients, and surgery followed by chemotherapy in the remaining one patient. In addition, 9 out of 11 patients with distant metastases at combined sites received chemotherapy (six patients with liver and lungs metastases and three patients with liver and peritoneal recurrences) while two remaining patients underwent surgery followed by chemotherapy (one patient with liver and peritoneal metastases and another patient with liver, lungs and bone metastases).

#### 3.2.2. Biochemical Markers

CEA provided a correct diagnosis in 65 patients, with true-positive results in 53 patients and true-negative results in 12 patients. Sixty-nine patients were misdiagnosed by CEA, including one patient with false-negative results and sixty-eight patients with false-positive results. CA 19-9 provided a correct diagnosis in 77 patients, with true-positive results in 23 patients and true-negative results in 54 patients. Fifty-six patients were misdiagnosed by CA 19-9, including thirty-one patients with false-negative results and twenty-six with false-positive results.

#### 3.2.3. Case Reports

**Figure 1:**
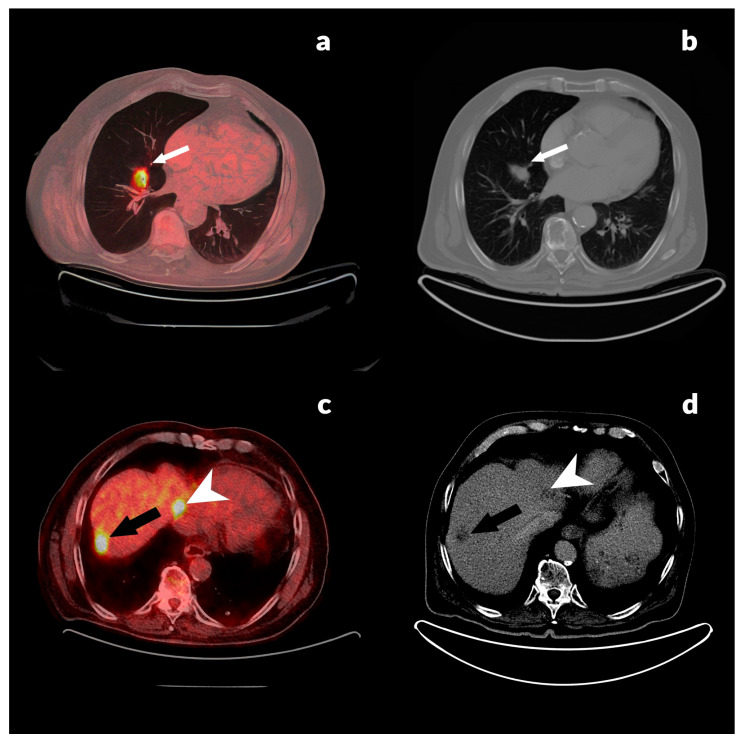
(**a**) PET/CT axial image detects a focal area of increased FDG uptake in the right pulmonary lobe, S4, SUVmax 7.50 (white arrow), corresponding to lung metastasis, (**b**) diagnostic CT scan in axial plane confirms a lesion in the right pulmonary lobe, S4 (white arrow), consistent with a metastatic lesion in the lung, (**c**) PET/CT axial image shows two hypermetabolic lesions in the liver in S8, SUVmax 8.9 (black arrow) and in S4a, SUVmax 8.0 (arrowhead), corresponding with liver metastases, and (**d**) diagnostic CT in axial plane shows two heterodense dominantly hypodense lesions in S8 (black arrow) and S4a (arrowhead), consistent with distant metastases in the liver.

**Figure 2:**
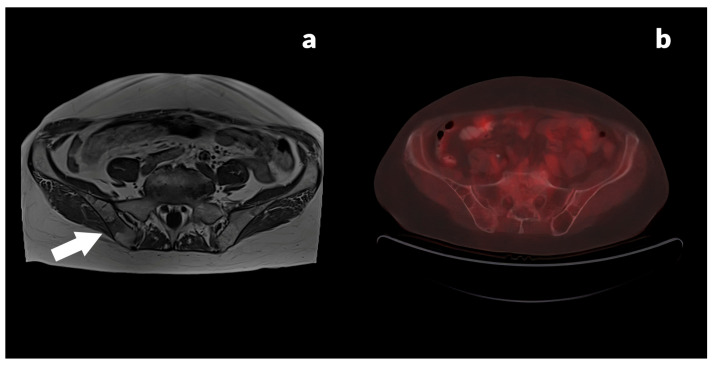
(**a**) Axial T1W MR image shows focal T1W hypointense lesion in the right iliac bone (white arrow) corresponding with metastasis, and (**b**) PET/CT axial image does not detect FDG-avid lesion in the region of pelvic bone.

**Figure 3:**
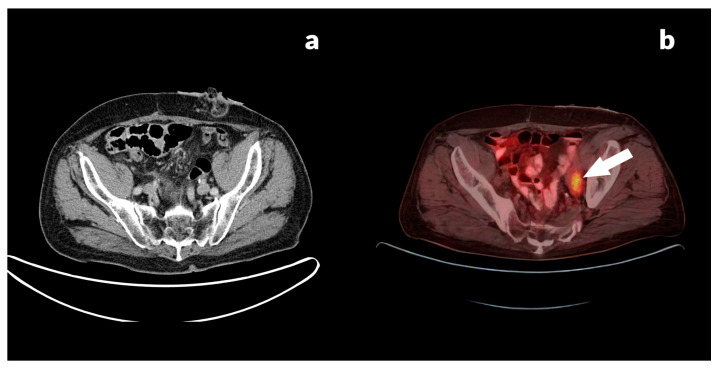
(**a**) Diagnostic CT in axial plane shows no lesion in the left pelvic region, and (**b**) PET/CT axial image detects an FDG-avid ovoid lesion in the left obturator area, SUVmax 7.20 (white arrow), corresponding with lymph node involvement.

CRC patients with confirmed recurrence who underwent both diagnostic imaging modalities, CECT and PET/CT, are presented in Figure 1, Figure 2 and Figure 3. Figure 1 shows an 88-year-old male with histopathologically confirmed diagnosis of rectal adenocarcinoma, pT3N2aM0 (differentiation tumor grade, G2), after a primary tumor resection followed by adjuvant therapy with elevated CEA level (12.6 ng/mL) and normal CA 19-9 level (2.5 U/mL).

Figure 2 shows a 70-year-old female with a histopathologically confirmed diagnosis of transversal colon adenocarcinoma, mucinous subtype, pT3N0M0 (differentiation tumor grade, G1), after surgical resection with elevated CEA level (21.0 ng/mL) and normal CA 19-9 level (2.0 U/mL).

Figure 3 presents an 84-year-old male with a rectal adenocarcinoma, pT3N2aM0 (differentiation tumor grade, G2), after completion of initial treatment (surgical resection and a subsequent adjuvant chemoradiation) with elevated CEA level (18.5 ng/mL) and normal CA 19.9 level (<0.6 U/mL).

### 3.3. Univariate Analyses

Univariate analyses showed that CRC recurrence was not significantly associated with gender (*p* = 0.211), mucinous tumor type (*p* = 0.158), presence of initial regional metastases (N+) (*p* = 0.583), and the presence of initial distant metastases (M+) (*p* = 0.201). Positive PET/CT scans are highly associated with CRC recurrence (*p*< 0.001) (Table 3).

No statistically significant difference was detected for CA 19-9 levels in patients with recurrence (*p* = 0.358). In contrast, patients without recurrent CRC (10.9 ± 20.4; median 7.56, IQR 4.7) show significantly lower CEA values (*p*< 0.001) than patients with recurrence (92.8 ± 268; median 19.9, IQR 29.2) (Table 4).

### 3.4. Diagnostic Efficacy Assessment

The sensitivity, specificity, PPV, NPV, and accuracy of PET/CT in detecting recurrent CRC are 94.4%, 82.5%, 78.5%, 95.7%, and 87.3%, respectively. CIM’s sensitivity, specificity, PPV, NPV, and accuracy in detecting recurrent CRC are 51.9%, 98.8%, 96.6%, 75.2%, and 79.9%, respectively. CEA’s sensitivity, specificity, PPV, NPV, and accuracy in detecting recurrent CRC were 98.1%, 15%, 43.8%, 92.3%, and 48.5%, respectively. The sensitivity, specificity, PPV, NPV, and accuracy of CA 19-9 in detecting recurrent CRC are 44.4%, 67.5%, 48%, 64.3%, and 58.2%, respectively (Table 5 and Table 6).

Table 6 shows the optimal cut-off values of CEA and CA 19-9 in detecting CRC recurrence.

**Figure 4:**
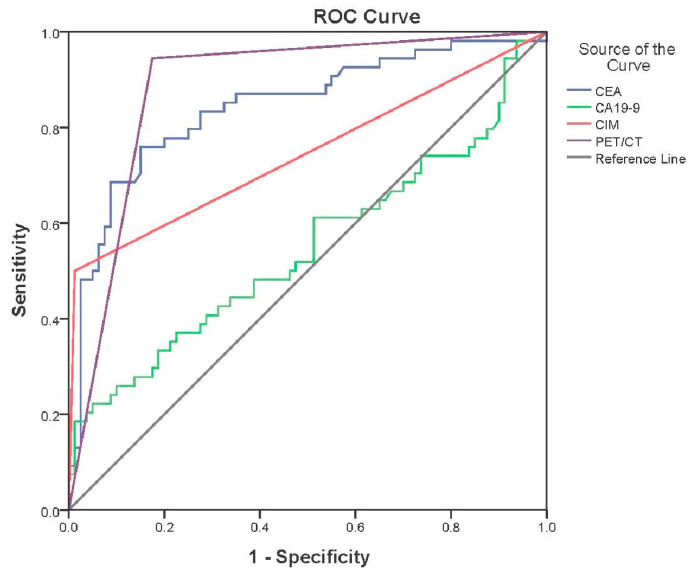
ROC analysis.

The ROC curve for PET/CT, CEA, CIM, and CA 19-9 was drawn (Figure 4). AUC (area under the curve) was 0.885 (95% CI: 0.824–0.946; *p* < 0.001) for PET/CT, 0.844 (95% CI: 0.772–0.916; *p* < 0.001) for elevated CEA levels, 0.753 (95% CI: 0.612–0.844; *p* < 0.001) for CIM, and 0.547 (95% CI: 0.442–0.652; *p* = 0.358) for elevated CA 19-9 levels.

### 3.5. Multivariate Analysis

Multivariate analysis (binary logistic regression model) was used to detect whether various demographic, clinical, and histological factors such as gender (male vs. female), CEA and CA 19-9 (normal values vs. elevated levels), histological tumor type (mucinous vs. non-mucinous), initial lymph node metastases (presence or absence), initial distant metastases (presence or absence), PET/CT results (positive vs. negative), and CIM results (positive vs. negative) were associated with the risk of recurrence during the follow-up of patients with colorectal disease. Results of the multivariate analysis showed that independent predictors for CRC recurrence were positive PET/CT scans (*p* < 0.001), positive CIM results (*p* = 0.001), and elevated CA19-9 levels (*p* = 0.023). Although CA 19-9 was not detected as a statistically significant predictor in the univariate analysis (*p* = 0.358), in a multivariate analysis, this tumor marker was recognized as a significant predicting factor for detecting CRC recurrence (*p* = 0.023) (Table 7).

## 4. Discussion

Since CRC often relapses after the initial treatment, regular patient follow-up is mandatory. Barreiro et al. reported a 5-year cumulative incidence of 13.7% recurrence with significantly increased incidence in rectal versus colon cancers (*p* = 0.024) and in advanced stage of disease (*p* < 0.001). In addition, they showed that recurrence has a negative impact on prognosis and outcome. Five-year disease-specific mortality increased from 3.8% to 33.6% in CRC recurrence-free patients one year after the surgery versus those who relapsed [9].

Early detection of recurrent CRC is essential for subsequent treatment (surgery, chemo, and/or radiation treatment). Serum markers, carcinoembryonic antigen (CEA), and carbohydrate antigen (CA19-9) have been studied in colorectal cancer for many years. CEA is a glycoprotein that is produced by normal fetal gut tissue and by epithelial tumors (specifically large bowel). As a tumor marker, CEA has been known since 1965 [25]. However, it is a non-specific biomarker and its increased levels are detected in malignant and benign conditions. CEA is mainly produced by colorectal, gastric, pancreatic, lung, ovarian, and breast cancers. It might also be elevated in different benign diseases, including chronic inflammatory bowel disease, diverticulitis, liver disease, and pancreatitis, as well as in conditions such as cigarette smoking and alcoholism [26,27,28]. CA 19-9 is a glycoprotein which has been in clinical practice since 1979 [29]. It is mostly used in the diagnosis of pancreatic carcinoma, colorectal cancers, and gastric neoplasm. Like CEA, CA 19-9 is not specific to certain histological types of tumors and organs of origin. An increased CA 19-9 level can also be found in several benign conditions, such as endometriosis, bronchiectasis, liver cirrhosis, or acute cholangitis [27,28,30]. There are numerous data on high CEA sensitivity in colorectal carcinoma, accounting for 65–74%, in contrast to much lower sensitivity for CA 19-9, which ranges from 26–48% only [28,31,32]. Measurements of CA 19-9 are not included in the standard follow-up of CRC patients due to its low sensitivity. Current guidelines include only CEA measurement along with colonoscopy, and standard imaging techniques (CT and MRI) in patients’ surveillance to improve recurrence detection [13,14,15]. Although CEA is well established, real-world medicine requires considering the local clinical settings. In our case, the institutional Tumor Board’s protocol recommends a routine 5-year surveillance of CRC patients with measurements of both CEA and CA 19-9. In fact, there are publications which indicate the role of CA 19-9 in detection of the recurrent CRC, especially if it is added to CEA measurements [17,28]. In addition, some authors reported increased CEA sensitivity if CA 19-9 measurements are combined with CEA levels [28,33,34,35]. Our results show that although CA 19-9 was not detected as a statistically significant predictor in the univariate analysis (*p* = 0.358), in a multivariable analysis, it was recognized as a significant predicting factor in detecting CRC recurrence (*p* = 0.023). Therefore, results would suggest that in clinical environments where CEA is not obtained for a variety of reasons CA 19-9 would be the alternative marker.

PET/CT is currently not considered the standard of care for the monitoring of CRC recurrence due to the insufficient evidence of its impact on patients’ management and outcome [36]. Current data on the role of PET/CT for recurrence detection in CRC patients’ surveillance were obtained mainly retrospectively and only a few prospective studies were yet performed. The retrospective studies showed inconsistent results of PET/CT diagnostic accuracy in detecting a CRC recurrence. While most of the studies detected high PET/CT diagnostic performance with 97–100% of sensitivity [37,38,39,40] and 91–97% of specificity [40,41,42], these values are much lower in other reports showing sensitivity and specificity of 86–95% [42,43,44,45] and 52–61%, respectively [37,44,46]. Well-designed randomized trials, including those on select patient populations and specifically defined surveillance protocols are necessary to establish the clinical impact of PET/CT imaging in the follow-up of CRC patients. However, due to the difficult nature of logistics required for such trials, robust prospective studies may be considered.

In terms of cost effectiveness, there is no doubt about the added value of PET/CT to the surveillance algorithm. Previous work by Buck et al. described in detail the key elements that are required for such analysis in their paper titled ”Economic Evaluation of PET and PET/CT in Oncology: Evidence and Methodologies approaches” [47]. Since our institutional Tumor Board’s protocol recommends PET/CT imaging in CRC patients with elevated tumor markers, we will simply state figures that may only provide one side of the whole cost story. The average cost of PET/CT imaging in our country is 470 EUR per patient and each patient may undergo about two PET/CT examinations annually. The monetary value on the use of PET/CT in these instances should never be considered in isolation. It should be stressed that limiting evaluation of a possible recurrence to conventional imaging (US, CT, and MRI) may induce additional unnecessary costs related to palliative treatment due to their limited accuracy. In addition, when considering the radiation impact of CECT, the use of PET/CT in a concept of “one stop shop” may in fact reduce the total cost of CRC recurrence evaluation.

A better CRC recurrence detection rate by PET/CT is reported in patients with increased CEA than in those with normal CEA levels [48,49]. Ozkan et al. detected higher specificity of PET/CT in CRC recurrence detection in patients with CEA of 10–14.9 ng/mL than in those with CEA levels of 5–9.9 ng/mL (75% versus 60%, respectively) [37]. Moreover, Vallam et al. showed that the likelihood of CRC recurrence depended on the rising CEA levels. The recurrent disease was detected in 10%, 45%, 70%, 94%, and 100% of patients if CEA levels were <5 ng/mL, 5.1–10 ng/mL, 10.1–15 ng/mL, 15.1–50 ng/mL, and >50 ng/mL, respectively [50].

In contrast to previous reports, several authors showed that increased CEA levels are not necessary for PET/CT in detecting recurrent CRC [38,39,45,51,52,53]. Miloradovic et al. reported sensitivity of 100% versus specificity of 83% for PET/CT in detecting recurrent CRC regardless of the CEA values [39]. Similarly, Sanli et al. did not find significant difference between efficacy of PET/CT in patients with normal and elevated CEA (sensitivity of 100% vs. 97.1% and specificity of 84% and 84.6%, respectively) [53]. In our study, we detected normal CEA levels in only one patient with a recurrence, while the remaining 53 patients with recurrent disease had an increased CEA. In contrast, 31 patients with recurrent CRC had normal CA 19-9, while an elevated CA 19-9 was found in 23 patients.

According to Sobhani et al., compared to conventional imaging, PET and PET/CT imaging ensure earlier detection of CRC recurrence during the patients’ surveillance without decreased recurrence rate [54,55]. Morphological imaging modalities (CT and MRI) cannot differentiate recurrence from post-operative inflammatory and post-irradiation changes in CRC patients. Since PET/CT provides information about the tumor metabolism, it helps in equivocal findings by distinguishing between a scar and a viable tumor [56,57,58,59]. In addition, PET/CT provides biological (functional) information that precedes morphological changes, resulting in malignant disease detection before it can be seen on CT and MRI [59].

In CRC patients with elevated CEA levels, several authors obtained better sensitivity and detection recurrence rates for PET/CT than conventional CT scans [37,44,60,61,62]. Comparing two imaging tools in detecting CRC recurrence in patients with elevated CEA, PET/CT shows a better detection rate of recurrence than CECT (71% vs. 55%) [60]. In addition, PET/CT showed higher sensitivity than MDCT (97.3% vs. 70.3%), with the same specificity for both techniques (94.4%) [61]. It has been reported that PET/CT was superior to CECT with reported better sensitivity (95–100% vs. 51–85%) and specificity (54–91% vs. 43–97%) [37,40,41,44]. In another study, Ince et al. compared diagnostic performance between PET/CT and CIM (CT or MRI) and detected better sensitivity and specificity for PET/CT (100% and 52%, respectively) than for CIM (71% and 87%, respectively) [46].

There are only a few studies performed on CRC patients with an aim to evaluate diagnostic efficacy of imaging modalities and tumor markers in detecting recurrence. Odalovic et al. compared diagnostic performance of PET/CT with MRI, CEA, and CA 19-9. They reported sensitivity, specificity, PPV, NPV, and accuracy of PET/CT of 92.6%, 75%, 92.6%, 75%, and 88.6%, and for MRI (65.4%, 66.7%, 85%, 40%, and 65.7%), respectively. In addition, CA 19-9 shows better overall accuracy in recurrence detection than CEA (48.6% and 54.3%, respectively) [62]. In a similar study performed on 75 patients, Uzun et al. reported that PET/CT was superior to CEA and CIM in detecting the CRC recurrent disease. They detected a sensitivity, specificity, accuracy, PPV, and NPV of 93.1%, 88.2%, 92%, 96.4%, and 78.9%, respectively for PET/CT, in contrast to these values of 72.4%, 64.7%, 70.6%, 87.5%, 40.7%, and 77.5%, 52.9%, 72%, 84.9%, and 40.8%, for CEA and CIM, respectively [21]. Caglar et al. compared the sensitivity and specificity of CEA and CA 19-9, CT, and PET/CT in 212 CRC patients with suspicious recurrent CRC. They reported low sensitivity for both CEA and CA 19-9 of 74% and 35%, and specificity of 86% and 83%; while these values for CT were 79% and 45% in detecting recurrence, respectively. PET/CT had superiority with sensitivity of 92% and specificity of 100%. The area under the ROC curve (AUC) of 0.865 and 0.631 was detected for CEA and CA 19.9, respectively. In addition, they detected the optimal cut-off serum levels for both CEA and CA 19-9, of ≥5.7 ng/L and ≥15.37 U/mL, with a sensitivity of 70.6% and 66.7% and a specificity of 94.4% and 66.7%, respectively [63]. In another study, Tan et al. suggested a cut-off of 2.2 μg/L for CEA which may yield the optimal balance of sensitivity and specificity in the detection of CRC recurrence [64].

Hancerliogullari et al. evaluated several risk factors for recurrent CRC. The strong predicting factors for recurrence detected in 59% of patients were initial lymph node metastases and SUVmax values [65].

In detecting recurrent CRC in our patients’ cohort, optimal cut-off values of 11.5 ng/mL for CEA and 120 U/mL for CA 19-9 were established. The sensitivity and specificity, according to the standard cut-off values for CEA and CA 19-9 (4.7 ng/l and 39 ng/mL respectively), were 98.1% and 15.0% for CEA, and 44.4% and 67.5% for CA 19-9, respectively. The sensitivity and specificity, according to the optimal cut-offs from ROC analyses obtained in our study (11.5 ng/l for CEA and 120 ng/mL for CA19-9), were 75.9% and 83.7% for CEA and 18.5% and 98.7% for CA19-9, respectively.

We did not find any significant difference in CA 19-9 levels in patients with recurrence compared to those without CRC recurrence (*p* = 0.358). However, the difference in CEA levels in patients with recurrent CRC versus patients without recurrence shows a statistically significant difference (92.8 ± 268; median 19.9, IQR 29.2, and 10.9 ± 20.4; median 7.56, IQR 4.7, respectively; *p* < 0.001).

According to the univariate analysis in our study, strong predicting factors for CRC recurrence are positive PET/CT (*p* < 0.001) and positive CIM results (*p* < 0.001). In contrast, gender (*p* = 0.211), mucinous component (*p* = 0.158), initial lymph node metastasis (N+) (*p* = 0.583), and initial distant metastasis (M+) (*p* = 0.201) were not significant predictors for recurrent CRC. In addition, multivariate analysis shows that independent predictors for CRC recurrence are positive PET/CT scans (*p*< 0.001), positive CIM scans (*p* = 0.001), and elevated CA19-9 levels (*p* = 0.023). Although CA 19-9 was not detected as a statistically significant predictor in the univariate analysis (*p* = 0.358), in a multivariate analysis, this tumor marker was recognized as a significant predicting factor in detecting a CRC recurrence (*p* = 0.023). The most accurate methods for CRC recurrence detection in our study are PET/CT (AUC of 0.885) and elevated CEA levels (AUC of 0.844). CIM shows a slightly lower value (AUC of 0.753), while CA 19-9 (AUC of 0.547) indicates a poor result. Additionally, a multivariate analysis indicates PET/CT as an independent predicting factor for recurrent disease (obtained by binary logistic regression, OR = 175). CEA is detected as a good recurrence predictor (AUC of 0.844; OR = 26).

Our results show that positive PET/CT made an impact on therapeutic decision making and altered treatment management in 26 recurrent CRC patients with false-negative CIM results (Chemotherapy was administered to 22 out of 26 patients, with only one having previously received excisional biopsy for local recurrence. Additionally, one patient underwent lung resection, two patients with liver metastases underwent surgery, and one patient with liver and peritoneal metastases received liver resection followed by chemotherapy).

It has been reported that PET/CT imaging has no role in mucinous CRC due to low or negligible FDG uptake influenced by a tumor hypocellularity and presence of mucin [66,67]. Some authors advocate that mucinous adenocarcinoma is the most common cause of false-negative PET/CT scans in patients with CRC recurrence [68,69]. Whiteford et al. reported a significantly lower sensitivity of PET/CT in mucinous versus non-mucinous tumor types (58% versus 92%, respectively; *p* = 0.05) [69]. Similar results were obtained by Berger et al. [70]. They detected a very low sensitivity for PET/CT in detecting mucinous CRC—false-negative results were detected in 41% of cases. They reported a significant correlation between PET failure to diagnose a recurrence with low tumor cellularity (*p* = 0.011) and overall abundance of mucin (*p* = 0.009). In our study, we detected only three false-negative PET/CT results—one patient had a mucinous adenocarcinoma. The PET/CT scan omitted small peritoneal lesions in one patient, probably due to scanner resolution limitation and a local recurrence in the third patient.

Colorectal cancer often metastasizes in the liver (in about 50–60%), with initial liver metastases accounting for approximately 30% [71]. Early detection of liver metastasis is of essential importance to allow for proper restaging and further patient selection for a liver resection. The MRI is the most sensitive diagnostic tool for detecting liver metastases [72]. In addition, PET/CT is also reported as a highly sensitive method in diagnostics of liver metastatic disease [73,74]. In comparison to CT, PET/CT is a superior diagnostic method, particularly in detecting occult metastases [38,48]. Results obtained in a study by Cinar et al. align with previous reports. They present PET/CT as more efficient than CT in detecting a CRC recurrence, with PET/CT having better sensitivity and specificity than CT (88% and 92% vs. 80% and 76%, respectively) [42].

Our results show that PET/CT has high accuracy in detecting distant metastases in the liver (consistent with MRI results in all cases) and lung metastases (having an agreement with CT scan only in 50% of cases due to false-negative CT results). Positive PET/CT results subsequently altered management in 26 patients with distant metastases—surgery was performed in eight patients (six patients with liver recurrences and two patients with lung metastases), surgery combined with chemotherapy in three patients (one patient with lung metastases, while the remaining two had combined metastatic disease including liver and peritoneal metastases in one patient and liver, lungs and bone metastases in another patient), and chemotherapy in 15 patients (including three patients with lung metastases and three patients with liver metastases, while nine patients had combined metastases including six patients with liver and lungs metastases and three patients with liver and peritoneal recurrences).

We obtained false-positive PET/CT results in 14 patients. Out of 14 patients, PET/CT scan was false-positive in 4 patients due to high FDG uptake in benign lesions—liver hemangioma was confirmed in two patients while lung granulomatous and lung inflammatory lesions were confirmed in the remaining two patients, respectively. Suspicious locoregional lesions in two patients and suspected regional lymph nodes in eight patients were misdiagnosed due to inflammatory changes in pelvic regions. It has been previously described in the literature that pelvic anatomy is altered in CRC patients as a result of surgery and radiation therapy, which might result in compromised findings due to false-positive results [56,75].

Our study has several limitations. PET/CT imaging is available in Serbia only at two Nuclear Medicine Centers, one of which is our institution. CRC patients suspected of having recurrence were referred from various centers across the country. CIM and tumor marker results were not standardized since they were obtained from different centers and laboratories. Another limitation of our study is that we did not provide the patients’ outcomes related to PET/CT findings. CRC patients were subsequently monitored and treated in their referring gastroenterology clinics that do not provide external access to their medical files. Moreover, the confirmation of CRC recurrence was obtained histopathologically in only 27.8% of patients, while in the remaining 72.2% by subsequent clinical and imaging follow-up. In addition, due to retrospective analysis of patients’ data, we could not change the diagnostic algorithm if necessary. Lastly, we did not classify patients into smokers and non-smokers, which may have had an impact on increased CEA levels and had been a bias in the determination of diagnostic performance. Another bias could be the patients’ selection and relatively short follow-up period. Finally, in view of the referral nature, data for autoimmune diseases would be logistically difficult to obtain. Patients who “potentially may have had autoimmune diseases” were not excluded and their underlying conditions might have affected the study.

## 5. Conclusions

F-18 FDG PET/CT shows a high diagnostic efficacy in detecting CRC recurrence. Compared to conventional imaging modalities, PET/CT shows a higher sensitivity and accuracy, but lower specificity in detecting the CRC recurrence in patients with elevated tumor markers. PET/CT should be routinely integrated into the post-operative follow-up of patients with CRC.

## Figures and Tables

**Table 1:** Patients’ demographic, clinical, and histopathological characteristics.

Demographic Characteristics	
Patients	134 (100%)
AGE	
Mean Males Females	69.6 ± 11.0 years 70.6 ± 11.3 years 68.2 ± 10.5 years
Range	39 89 years
Gender	
Males	79 (59%)
Females	55 (41%)
TNM Staging	
T Stage	
T1	1 (0.75%)
T2	11 (8.21%)
T3	102 (76.12%)
T4	20 (14.92%)
N Stage	
N0	56 (41.79%)
N1	50 (37.31%)
N1a	38 (28.36%)
N1b	9 (6.72%)
N1c	3 (2.23%)
N2	28 (20.90%)
N2a	17 (12.69%)
N2b	11 (8.21%)
M Stage	
M0	122 (93.28%)
M1	9 (6.72%)
Tumor Localization	
ascending colon	17 (12.69%)
Caecum	11 (8.21%)
transversal colon	12 (8.95%)
descending colon	10 (7.46%)
Sigmoid	25 (18.65%)
Rectosigmoid	13 (9.70%)
Rectum	35 (26.12%)
Splenic flexure	6 (4.48%)
Liver flexure	3 (2.24%)
Duplex (caecum + ascending)	1 (0.75%)
Duplex (transversal + descending)	1 (0.75%)
Tumor Histology and Differentiation Grade	
Non-mucious adenocarcinoma	112 (83.58%)
Well differentiated—G1	16 (11.94%)
Moderately differentiated—G2	81 (60.45%)
Poorly differentiated—G3	15 (11.19%)
Mucinous adenocarcinoma	22 (16.42%)
Initial Treatment	
Surgery only	19 (14.18%)
Surgery + chemotherapy	8 (5.97%)
Neoadjuvant chemoradiation + surgical resection	4 (2.98%)
Neoadjuvant chemoradiation+surgical resection+ chemotherapy	11 (8.21%)
Surgical resection + Adjuvant chemotherapy	86 (64.18%)
Surgical resection + Adjuvant chemoradiation	5 (3.73%)
Surgical resection + Adjuvant radiation therapy	1 (0.75%)

**Table 2:** Patients with CRC recurrence.

Site of Recurrence (No of Patients)	Method of Recurrence Confirmation		
	Histology (15)		Follow-Up (39)
	Operation (13)	Biopsy (2)	
Local (7)	2	1	4
Regional lymph nodes (11)	0	0	11
Locoregional (4)	0	0	4
Peritoneum (5)	0	0	5
Liver (9)	6	0	3
Lungs (6)	3	0	3
Bone (1)	0	0	1
Combined sites			
Liver + lungs (6)	0	1	5
Liver + peritoneum (4)	1	0	3
Liver+ lungs+ bone (1)	1	0	0

**Table 3:** The univariate analysis of CRC recurrence predicting factors (Fisher’s exact test).

Factor	Whole Population (*n* = 134)	CRC Recurrence (*n* = 54)	Remission (*n* = 80)	*p*-Value
Male gender (%)	79 (59.0%)	28 (51.9%)	51 (63.8%)	0.211
Mucinous type	22 (16.4%)	12 (22.2%)	10 (12.5%)	0.158
N (+)	77 (57.5%)	33 (61.1%)	44 (55.0%)	0.583
M (+)	10 (7.5%)	6 (11.1%)	4 (5.0%)	0.201
FDG PET/CT (+)	65 (48.5%)	51 (94.4%)	14 (17.5%)	< 0.001
CIM (+)	28 (20.9%)	27 (50.0%)	1 (1.2%)	< 0.001

Male gender; mucinous tumor type; N (+), presence of initial regional metastases; M (+), presence of initial distant metastases; FDG PET/CT (+), positive PET/CT findings; CIM (+), positive CIM findings.

**Table 4:** Serum CEA (ng/mL) and CA 19-9 (U/mL) levels in CRC patients (Mann–Whitney U test).

Test	Recurrence	n = 134	Mean	Median	Minimum	Maximum	*p*-Value
CEA	Yes	54	92.8	19.9	0.9	1624	<0.001
	No	80	10.9	7.56	1.3	178	
CA 19-9	Yes	54	88.6	18.8	0.5	1637	0.358
	No	80	30.7	17.0	0.5	194	

**Table 5:** Diagnostic efficacy of PET/CT, CIM, CEA, and CA 19-9 in CRC recurrence detection.

Diagnostic Method	%	95% CI (Confidence Interval)	
**PET/CT**			
SN	94.4	91.2	97.1
SP	82.5	77.1	87.9
PPV	78.5	72.6	84.3
NPV	95.7	92.8	98.5
FPV	21.5	15.7	27.4
FNV	4.3	0.0	7.2
ACC	87.3	82.6	92.0
**CIM**			
SN	51.9	44.8	58.9
SP	98.8	97.2	100.0
PPV	96.6	94.0	99.1
NPV	75.2	69.1	81.4
FPV	3.4	0.9	6.0
FNV	24.8	18.6	30.9
ACC	79.9	74.2	85.5
**CEA**			
SN	98.1	96.2	100.0
SP	15.0	9.9	20.1
PPV	43.8	36.8	50.8
NPV	92.3	88.5	96.1
FPV	56.2	49.2	63.2
FNV	7.7	3.9	11.5
ACC	48.5	41.4	55.6
**CA 19-9**			
SN	44.4	37.4	51.5
SP	67.5	60.8	74.2
PPV	48.0	40.9	55.1
NPV	64.3	57.5	71.1
FPV	52.0	44.9	59.1
FNV	35.7	28.9	42.5
ACC	58.2	51.2	65.2

**Table 6:** Optimal cut-off values of CEA and CA 19-9 in CRC recurrence detection.

	Sensitivity (%)	Specificity (%)
FDG PET/CT	94.4	82.5
CIM	51.9	98.8
CEA normal < 4.7 ng/L	98.1	15.0
CEA normal < 11.5 ng/L *	75.9	83.7
CA 19-9 normal < 39 ng/L	44.4	67.5
CA 19-9 normal < 120 ng/L *	18.5	98.7

* Optimal cut-off from our study.

**Table 7:** Multivariate analysis of CRC recurrence predicting factors (overall percentage 90.3%).

Factor	B	*p*	Exp (B)
CEA (+)	5.634	0.107	279
CA 19-9 (+)	2.553	0.023	12.8
CIM (+)	5.759	0.001	317
FDG PET/CT (+)	5.637	<0.001	280
Constant	−11.090	0.007	0.00

FDG PET/CT (+), positive PET/CT scan; CIM (+), positive CT scan; CA 19-9 (+), elevated CA 19-9 levels; CEA (+), elevated CEA levels.

## Data Availability

The data presented in this study are available on request from the corresponding author. The data are not publicly available due to patients’ privacy.
